# Corrigendum: EEG-based spectral analysis showing brainwave changes related to modulating progressive fatigue during a prolonged intermittent motor task

**DOI:** 10.3389/fnhum.2022.958768

**Published:** 2022-08-17

**Authors:** Easter S. Suviseshamuthu, Vikram Shenoy Handiru, Didier Allexandre, Armand Hoxha, Soha Saleh, Guang H. Yue

**Affiliations:** ^1^Center for Mobility and Rehabilitation Engineering Research, Kessler Foundation, West Orange, NJ, United States; ^2^Department of Physical Medicine and Rehabilitation, Rutgers Biomedical Health Sciences, Newark, NJ, United States

**Keywords:** EEG, fatigue, maximum voluntary contraction, motor cortex, power spectral density, submaximal muscle contraction, time-frequency analysis, elbow flexion

In the published article, there was an error. **Equation (2) is incorrect**.

A correction has been made to **Data Analysis**, ***Statistical Framework***, **Paragraph Two**. This sentence previously stated: Therefore, the ERSP analyses and the results reported here are based on the log-transformed ERSP in Equation (2) and its time-averaged quantity in Equation (3), both expressed in decibels (dBs):

(2) $$\begin{array}{r} \text{ERSP}\left(f,t\right)=10\log_{10}\left[\text{ERS}\left(f,t\right)-{\text{B}}_{\text{AVG}}\left(f\right)\right] \end{array}$$

(3) $$\tilde{\text{ERSP}}(f)=\frac{1}{r}\sum_{\underline{t}\in R}\text{ERSP}(f,\underline{t})$$

where

(4) $$\begin{array}{r} B_{\text{AVG}}\left(f\right)=\frac{1}{pq}\overset{p}{\underset{k=1}{\sum }}\underset{\bar{t}\in Q}{\sum }{\left|S_{k}\left(f,\bar{t}\right)\right|}^{2} \end{array}$$

and *r* denotes the cardinality of the set *R* of steady- or post-contraction time points.

The corrected sentence appears below: Therefore, the ERSP analyses and the results reported here are based on the log-transformed ERSP in Equation (2) and its time-averaged quantity in Equation (3), both expressed in decibels (dBs):

(2) $$\begin{array}{r} \text{ERSP}\left(f,t\right)=10\log_{10}\left[\text{ERS}\left(f,t\right)⊘{\text{B}}_{\text{AVG}}\left(f\right)\right] \end{array}$$

(3) $$\tilde{\text{ERSP}}(f)\text{​​​​​​​​​​​​​ }=\frac{1}{r}\sum_{\underline{t}\in R}\text{ERSP}(f,\underline{t})$$

where

(4) $$\begin{array}{r} B_{\text{AVG}}\left(f\right)=\frac{1}{pq}\overset{p}{\underset{k=1}{\sum }}\underset{\bar{t}\in Q}{\sum }{\left|S_{k}\left(f,\bar{t}\right)\right|}^{2} \end{array}$$

⊘ denotes Hadamard division, and *r* is the cardinality of the set *R* of steady- or post-contraction time points.

The authors apologize for this error and state that this does not change the scientific conclusions of the article in any way. The original article has been updated.

## Publisher's note

All claims expressed in this article are solely those of the authors and do not necessarily represent those of their affiliated organizations, or those of the publisher, the editors and the reviewers. Any product that may be evaluated in this article, or claim that may be made by its manufacturer, is not guaranteed or endorsed by the publisher.

