# Supplementary material for: EEG-Based Spectral Analysis Showing Brainwave Changes Related to Modulating Progressive Fatigue During a Prolonged Intermittent Motor Task
Source: Front Hum Neurosci. 2022 Mar 11;16:770053. doi: 10.3389/fnhum.2022.770053 (PMC8962200; doi:10.3389/fnhum.2022.770053)
Supplement: Supplementary file 1 [file Data_Sheet_1.PDF]

Supplementary Material

1 SUPPLEMENTARY TABLE

Table S1. Demographics of the healthy individuals, who participated in the study to perform an intermittent submaximal elbow flexion task until self-perceived exhaustion. BMI and BFI stand for body mass index and brief fatigue inventory, respectively.

Subj.	Age (yrs.)	Gender	BMI	Endurance (s)	BFI
1	46	M	28.7	1862	0.0
2	58	F	32.6	1792	0.0
3	44	M	30.0	1852	0.4
4	[26, 48]	F	NA	1882	NA
5	26	M	35.3	2183	0.0
6	56	F	24.8	731	1.7
7	59	F	33.5	1934	0.7
8	70	M	25.1	1893	2.6
9	48	F	22.3	1936	1.0
10	36	M	NA	1981	NA
11	72	M	47.9	1905	0.9
12	55	F	28.3	1951	0.1
13	58	F	26.0	1918	3.0
14	48	F	22.2	1864	0.6

NOTE: The entries denoted as “NA” implies that the detail is not available. For subject 4, the exact age information is not available. However, the range can be specified based on the median age of the cohort mentioned in Cai et al., 2014. BFI is calculated as described in the following article: Mendoza, T. R., Wang, X. S., Cleeland, C. S., Morrissey, M., Johnson, B. A., Wendt, J. K., and Huber, S. L. (1999). The rapid assessment of fatigue severity in cancer patients: Use of the Brief Fatigue Inventory. *Cancer*, 85(5), 1186–1196.

2 SUPPLEMENTARY FIGURES

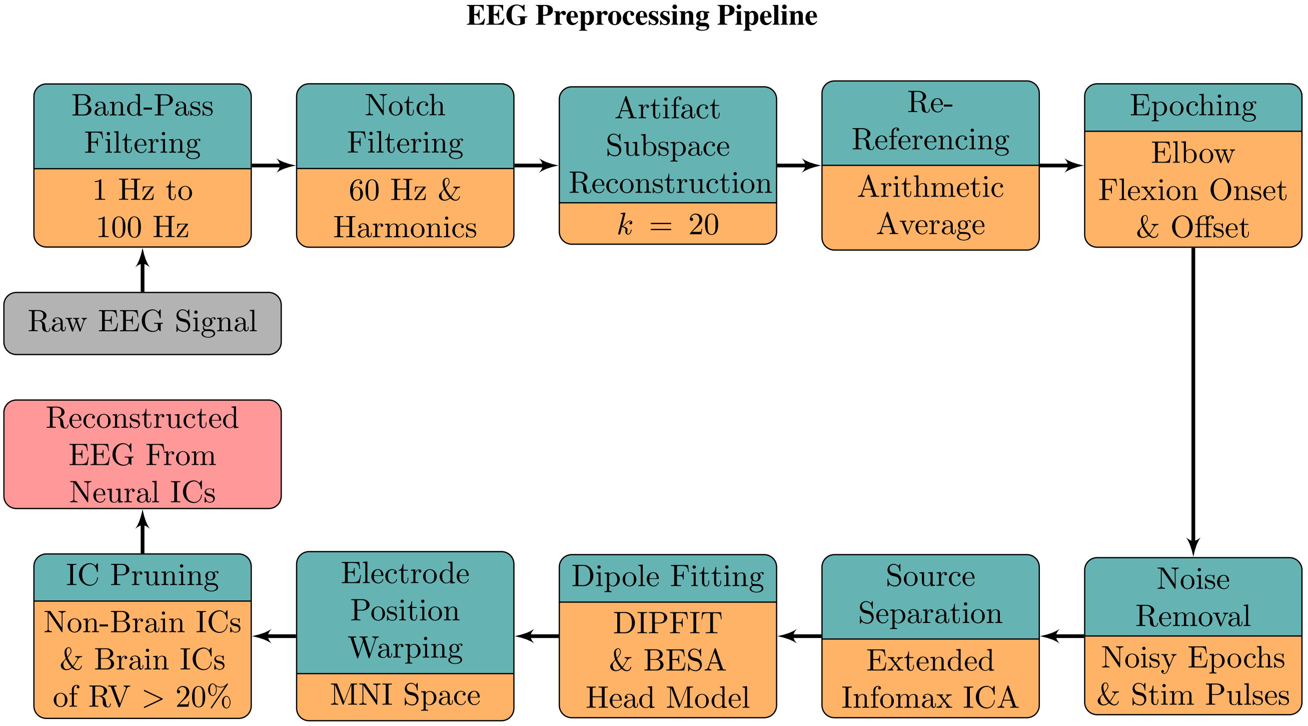

Figure S1. The raw EEG signals (gray) underwent the preprocessing steps (teal) in the following sequence: band-pass filtering, notch filtering, ASR, re-referencing, epoching, noisy epoch removal, blind source separation, dipole fitting, electrode position warping, and IC pruning. The parameter choice or specifications (orange) related to each procedure are shown inside the blocks. The brain-related ICs were used to reconstruct the ICA-cleaned EEG signals (red) for performing the ERSP analyses.

Framework for Conducting Statistical Inferential Tests on PSD Values at Group Level

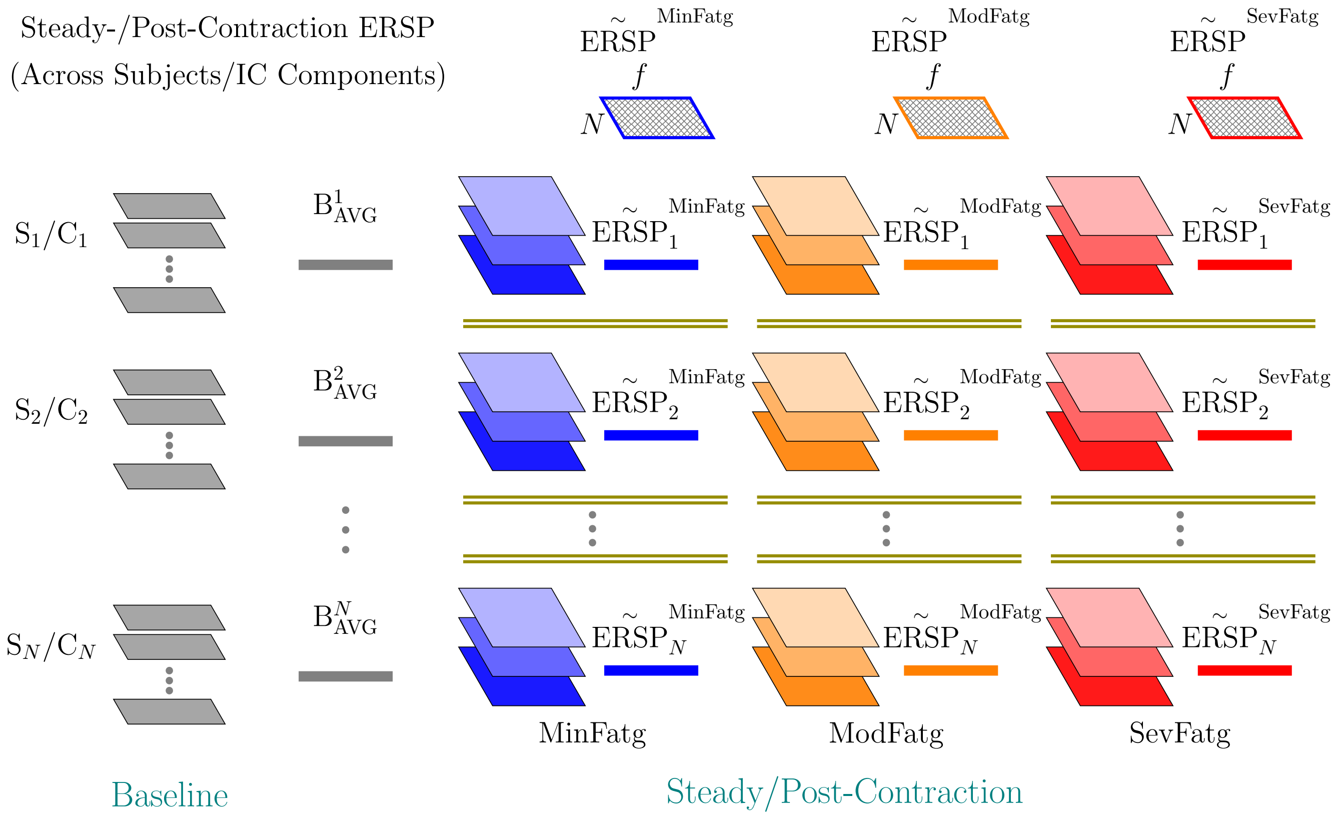

Figure S2. The framework for conducting statistical inferential tests to investigate whether the ERSP of steady contraction or post-contraction computed at the channel- or source-level for an EEG frequency band of interest scales with the fatigue level. The common baseline $\mathrm{B}^j_{\mathrm{AVG}}$ (thick gray line in row j) was derived for the j-th subject (or component) by averaging the baseline power spectra (gray parallelograms in row j) across the trials, time points, and fatigue conditions. The stacked color parallelograms in the j-th row denote the data power spectra $|S_k(f,t)|^2$, $k = 1, 2, \ldots, M$, for the j-th subject (or component) under a fatigue condition, where f and t represent the frequency and time points, respectively. The color of a parallelogram signifies the fatigue condition—blue, orange, and red stand for $\mathrm{MinFatg}$, $\mathrm{ModFatg}$, and $\mathrm{SevFatg}$, respectively. For the mean of M data power spectra of each subject, the log-transformed ERSPs were computed using (2), which were then averaged across the steady- or post-contraction time points and denoted as $\widetilde{\mathrm{ERSP}}_j^{\mathrm{FatgCond}}$ in (3) for the j-th subject (or component) with $\mathrm{FatgCond} \in \{\mathrm{MinFatg}, \mathrm{ModFatg}, \mathrm{SevFatg}\}$ (thick line with the respective color). By vertically concatenating N row vectors $\widetilde{\mathrm{ERSP}}_j^{\mathrm{FatgCond}}$ as in (5), a matrix represented as $\widetilde{\mathrm{ERSP}}^{\mathrm{FatgCond}}$ was constructed and depicted using a crosshatch-patterned parallelogram with white background and a color border. Each subject S_j (channel-level) or component C_j (source-level) is isolated from the adjacent ones by a pair of horizontal lines (olive green). Note that the number of subjects N is assumed to be equal to the number of components in an IC cluster for simplicity, but it is not necessarily the case.

Two- & Three-Class Linear SVM Classification: Confusion Matrices

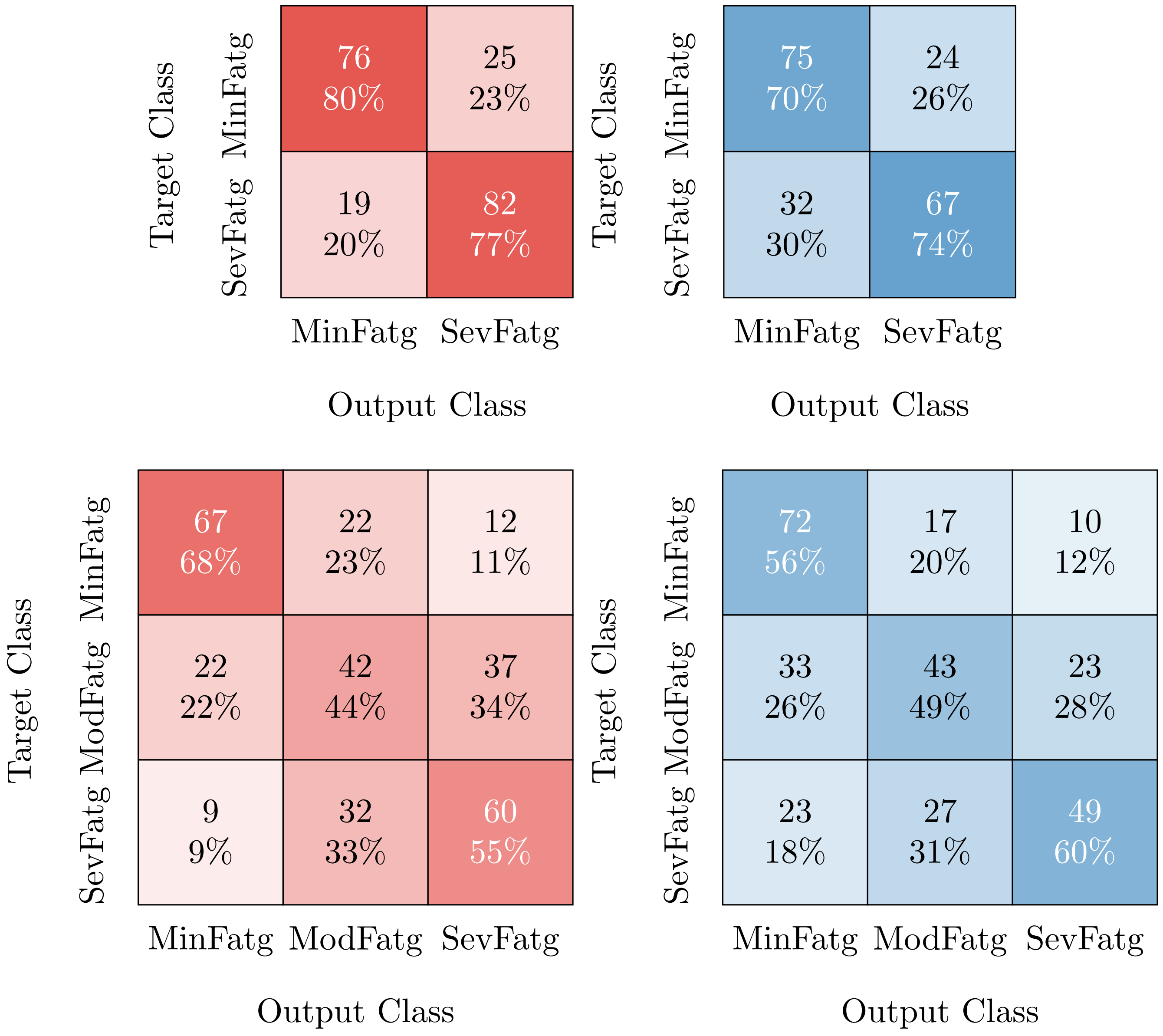

Figure S3. 12-D feature vectors comprising the ERSP values of individual IMT trials from six EEG channels (FC3, C1, C3, C5, CP1, and CP3) and two frequency bands (alpha and beta) were supplied to a linear SVM (LSVM) classifier for classifying the fatigue levels. The outcome of LSVM from a 10-fold cross validation is represented as confusion matrices of the binary—MinFatg and SevFatg—(row 1) and three-class classification (row 2) for the steady-contraction (left panel) and post-contraction EEG data (right panel).

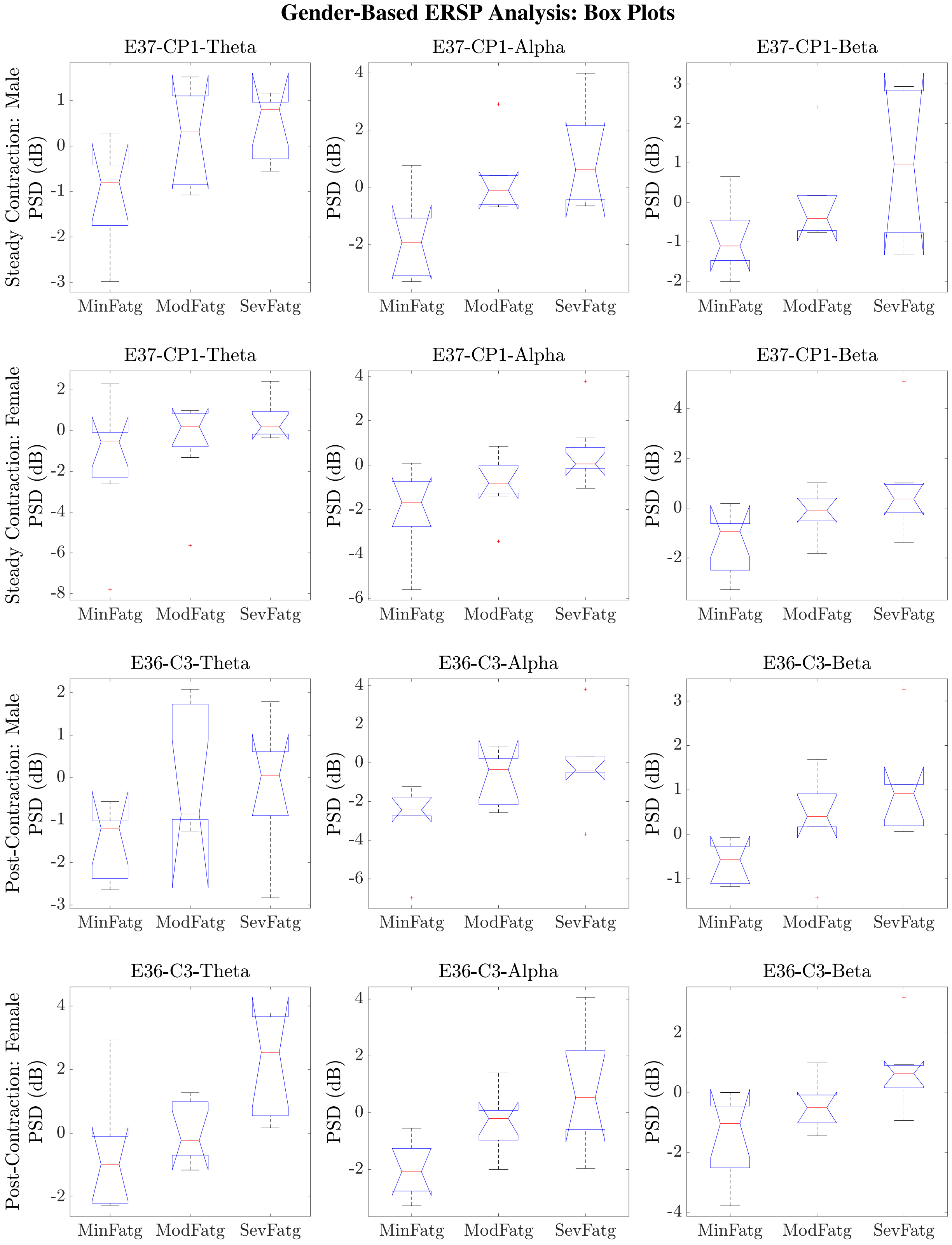

Figure S4. The box plots of $\overset{\approx}{\text{ERSP}}^{\text{FatgCond}}$ to demonstrate that the relationship between the PSD and fatigue is preserved, albeit separate channel-level analysis with male and female subjects. For a representative channel CP1, the ERSP box plots for steady-contraction EEG data from the male and female subjects are displayed in row 1 and 2, respectively. Similarly, for the post-contraction data from channel C3, the results corresponding to the male and female subjects are presented in row 3 and 4, respectively.

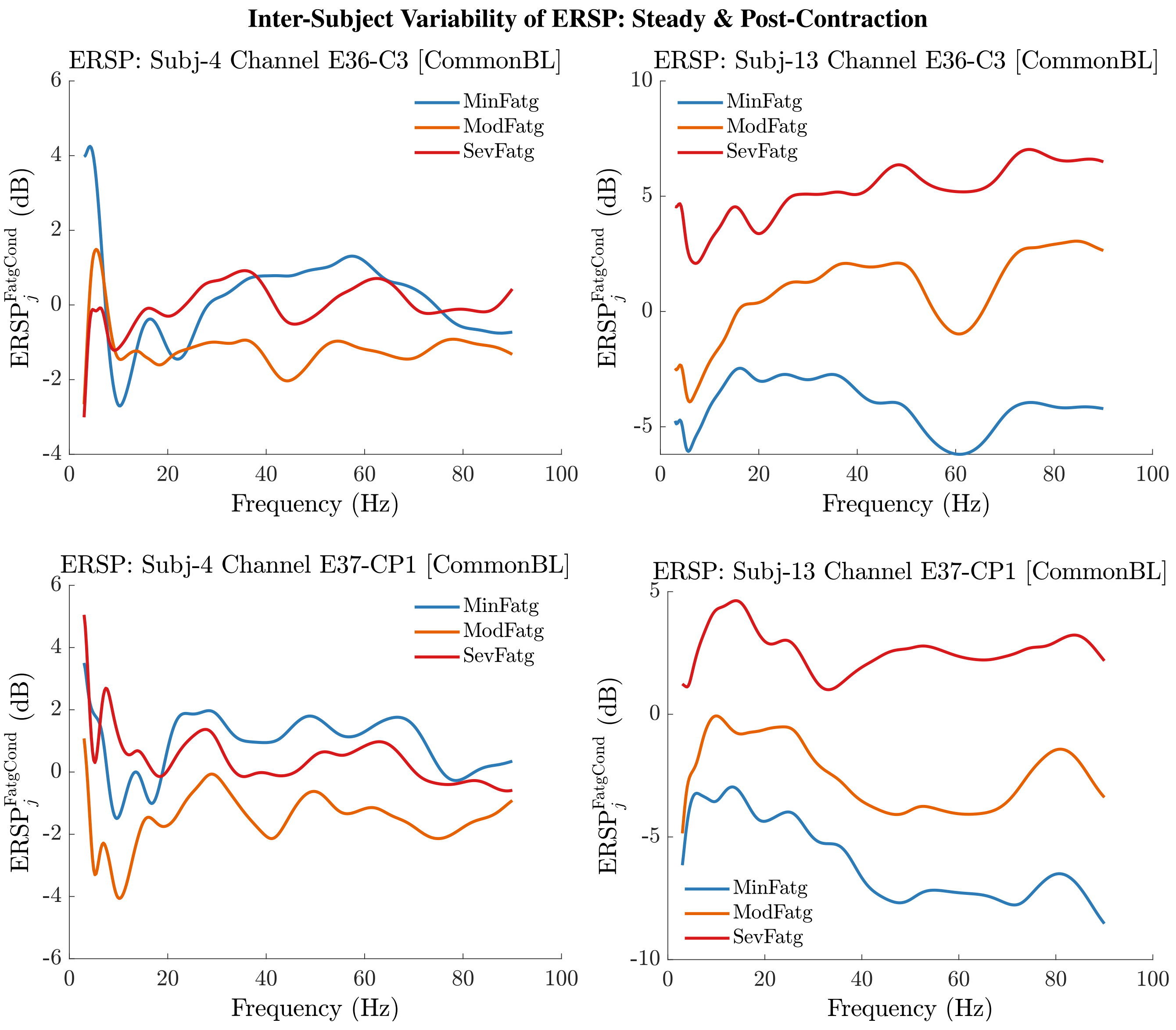

Figure S5. The $\widetilde{\mathrm{ERSP}}_j^{\mathrm{FatgCond}}$ plots were generated for channel C3 and CP1 by averaging $\mathrm{ERSP}_j^{\mathrm{FatgCond}}$ over the steady-contraction (1st row) and post-contraction time interval (2nd row), respectively, for two subjects to illustrate the inter-subject ERSP variability. For an outlier case [Subj-4 (left panel)], both the ERSP plots reveal that the PSD does not vary in proportion to fatigue within the theta, alpha, and beta frequency bands. Whereas, in a typical case [Subj-13 (right panel)], the PSD averaged over these time intervals increases monotonically with the perceived fatigue for all EEG frequency bands.
